# 1,10-Phenanthrolinium 2′-carboxy­biphenyl-2-carboxyl­ate

**DOI:** 10.1107/S1600536809036551

**Published:** 2009-09-16

**Authors:** Chongchen Wang

**Affiliations:** a School of Environmental and Energy Engineering, Beijing University of Civil Engineering and Architecture, 100044 Beijing, People’s Republic of China

## Abstract

The title complex, C_12_H_9_N_2_^+^·C_14_H_9_O_4_^−^ or (Hphen)^+^(Hbptc)^−^ [H_2_btc = 2,2′-biphenyl­dicarboxylic acid and phen = 1,10-phenanthroline], has been synthesized under hydro­thermal conditions. The compound is composed of discrete cations (Hphen)^+^ and anions (Hbptc)^−^, which are linked by electrovalent bonding; the molecular and crystal structures are further strengthened by intra­molecular O—H⋯O and inter­molecular N—H⋯O hydrogen bonds.

## Related literature

For the applications of the metal-organic coordination compounds constructed from biphenydicarboxylic acid, see: Gao & Cheng (2004 ▶).
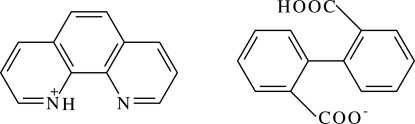


## Experimental

### 

#### Crystal data

C_12_H_9_N_2_^+^·C_14_H_9_O_4_^−^
*M* *_r_* = 422.42
Monoclinic, 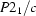

*a* = 12.1661 (13) Å
*b* = 7.389 (1) Å
*c* = 22.160 (2) Å
β = 91.061 (1)°
*V* = 1991.8 (4) Å^3^
*Z* = 4
Mo *K*α radiation
μ = 0.10 mm^−1^
*T* = 298 K
0.48 × 0.45 × 0.37 mm

#### Data collection

Bruker SMART CCD area detector diffractometer
Absorption correction: multi-scan (*SADABS*; Bruker, 2007 ▶) *T*_min_ = 0.955, *T*_max_ = 0.965
9415 measured reflections
3479 independent reflections
1771 reflections with *I* > 2σ(*I*)
*R*_int_ = 0.102

#### Refinement

*R*[*F* ^2^ > 2σ(*F* ^2^)] = 0.076
*wR*(*F* ^2^) = 0.243
*S* = 1.01
3479 reflections
289 parameters
H-atom parameters constrained
Δρ_max_ = 0.30 e Å^−3^
Δρ_min_ = −0.29 e Å^−3^

### 

Data collection: *SMART* (Bruker, 2007 ▶); cell refinement: *SAINT* (Bruker, 2007 ▶); data reduction: *SAINT*; program(s) used to solve structure: *SHELXS97* (Sheldrick, 2008 ▶); program(s) used to refine structure: *SHELXL97* (Sheldrick, 2008 ▶); molecular graphics: *SHELXTL* (Sheldrick, 2008 ▶); software used to prepare material for publication: *SHELXTL*.

## Supplementary Material

Crystal structure: contains datablocks global, I. DOI: 10.1107/S1600536809036551/ez2179sup1.cif

Structure factors: contains datablocks I. DOI: 10.1107/S1600536809036551/ez2179Isup2.hkl

Additional supplementary materials:  crystallographic information; 3D view; checkCIF report

## Figures and Tables

**Table 1:** Hydrogen-bond geometry (Å, °)

*D*—H⋯*A*	*D*—H	H⋯*A*	*D*⋯*A*	*D*—H⋯*A*
N1—H1⋯O1^i^	0.86	1.82	2.643 (4)	161
O3—H3⋯O1	0.82	1.75	2.559 (4)	172

Symmetry code: (i) 
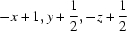
.

**Fig. 1.**
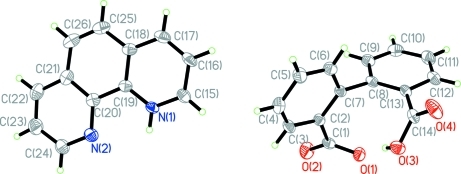
ORTEP drawing of the title compound showing the atom labelling scheme. Ellipsoids are drawn at the 30% level.

**Fig. 2.**
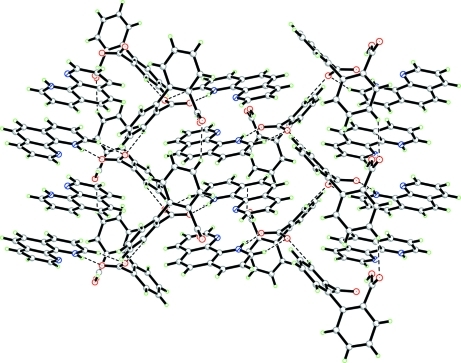
The crystal packing of the title complex. Hydrogen bonds are indicated by dashed lines.

## supplementary crystallographic information

### Comment

1,10-phenanthroline and 2,2'-biphenyldicarboxylic acid are both versatile ligands, which have been used in the self-assembly of various coordination compounds. The crystals of the title compound were obtained unintentionally as the harvested product of the hydrothermal reaction between Ce_2_O_3_ and 1,10-phenanthroline in the presence of 2,2'-biphenyldicarboxylic acid.

In the crystal of the title compound, 1,10-phenanthroline is protonated into the cation 1,10-phenanthrolinium [(Hphen)^+^], as indicated by the presence of a peak of electron density on the difference Fourier map at an appropriate distance from N1. The 2,2'-biphenydicarboxylic acid has been deprotonated into the anion biphenyldicarboxylate [(Hbptc)^-^], with the similar C—O distances on the deprotonated carboxylate indicating delocalisation (O1—C1=1.274 (5); O2—C1=1.221 (5)Å), whereas the C—O distances in the carboxylic acid group (O3—C14=1.315 (5); O4—C14=1.197 (5) Å) correspond to single and double bonds, respectively. The discrete cations (Hphen)^+^ and anions (Hbptc)^-^ are linked by an electrovalent bond, and further strengthened by intermolecular N1—H1···O1^i^(Symmetry operator, i: -*x* + 1, *y* + 1/2, -*z* + 1/2)  and intramolecular O3—H3···O1 hydrogen bonds.

The phenyl rings of the 2,2'-biphenyldicarboxylate are not coplanar, but twisted almost perpendicular to each other with a dihedral angle between the planes through the two rings of 86.06(0.13) °.

### Experimental

The mixture of 2,2'-biphenydicarboxylic acid (0.04884 g), 1,10-phenanthroline (0.0360 g), Ce_2_O_3_ (0.0702 g) and deionized water (15 ml) was heated in a 23 ml teflon-lined reaction vessel at 433 K for 120 h to carry out the hydrothermal reaction. After slow cooling to room temperature the mixture was filtered, and the clear solution obtained was allowed to evaporate slowly at room temperature. After about 10 days, yellow block-like crystals of the title compound were harvested.

### Refinement

The positions of the hydrogen atoms on O3 and N1 were identified from a difference Fourier map. All H atoms were then fixed geometrically and allowed to ride on their parent carbon atoms, with C—H distances of 0.93 Å, an N—H distance of 0.86 Å and an O—H distance of 0.82 Å; and with *U*_iso_(H) =  1.2*U*_eq_(C, N) or 1.5*U*_eq_(O).

### Crystal data

**Table:**

C_12_H_9_N_2_^+^·C_14_H_9_O_4_^−^	*F*(000) = 880
*M**_r_* = 422.42	*D*_x_ = 1.409 Mg m^−^^3^
Monoclinic, *P*2_1_/*c*	Mo *K*α radiation, λ = 0.71073 Å
*a* = 12.1661 (13) Å	Cell parameters from 1541 reflections
*b* = 7.389 (1) Å	θ = 2.5–21.4°
*c* = 22.160 (2) Å	µ = 0.10 mm^−^^1^
β = 91.061 (1)°	*T* = 298 K
*V* = 1991.8 (4) Å^3^	Block, brown
*Z* = 4	0.48 × 0.45 × 0.37 mm

### Data collection

**Table:**

Bruker SMART CCD area detector diffractometer	3479 independent reflections
Radiation source: fine-focus sealed tube	1771 reflections with *I* > 2σ(*I*)
graphite	*R*_int_ = 0.102
phi and ω scans	θ_max_ = 25.0°, θ_min_ = 1.7°
Absorption correction: multi-scan (*SADABS*; Bruker, 2007)	*h* = −14→14
*T*_min_ = 0.955, *T*_max_ = 0.965	*k* = −8→7
9415 measured reflections	*l* = −22→26

### Refinement

**Table:**

Refinement on *F*^2^	Primary atom site location: structure-invariant direct methods
Least-squares matrix: full	Secondary atom site location: difference Fourier map
*R*[*F*^2^ > 2σ(*F*^2^)] = 0.076	Hydrogen site location: inferred from neighbouring sites
*wR*(*F*^2^) = 0.243	H-atom parameters constrained
*S* = 1.01	*w* = 1/[σ^2^(*F*_o_^2^) + (0.0849*P*)^2^ + 0.7223*P*] where *P* = (*F*_o_^2^ + 2*F*_c_^2^)/3
3479 reflections	(Δ/σ)_max_ < 0.001
289 parameters	Δρ_max_ = 0.30 e Å^−^^3^
0 restraints	Δρ_min_ = −0.29 e Å^−^^3^

### Special details

**Table:**

Geometry. All e.s.d.'s (except the e.s.d. in the dihedral angle between two l.s. planes) are estimated using the full covariance matrix. The cell e.s.d.'s are taken into account individually in the estimation of e.s.d.'s in distances, angles and torsion angles; correlations between e.s.d.'s in cell parameters are only used when they are defined by crystal symmetry. An approximate (isotropic) treatment of cell e.s.d.'s is used for estimating e.s.d.'s involving l.s. planes.
Refinement. Refinement of *F*^2^ against ALL reflections. The weighted *R*-factor *wR* and goodness of fit *S* are based on *F*^2^, conventional *R*-factors *R* are based on *F*, with *F* set to zero for negative *F*^2^. The threshold expression of *F*^2^ > σ(*F*^2^) is used only for calculating *R*-factors(gt) *etc*. and is not relevant to the choice of reflections for refinement. *R*-factors based on *F*^2^ are statistically about twice as large as those based on *F*, and *R*- factors based on ALL data will be even larger.

### Fractional atomic coordinates and isotropic or equivalent isotropic displacement parameters (Å^2^)

**Table:**

	*x*	*y*	*z*	*U*_iso_*/*U*_eq_	
N1	0.4893 (3)	0.7686 (4)	0.46216 (17)	0.0382 (9)	
H1	0.5462	0.8170	0.4464	0.046*	
N2	0.6719 (3)	0.8548 (5)	0.52863 (18)	0.0486 (11)	
O1	0.3597 (2)	0.4014 (4)	0.10974 (14)	0.0472 (8)	
O2	0.4735 (2)	0.3641 (5)	0.18797 (16)	0.0674 (11)	
O3	0.1925 (2)	0.5887 (4)	0.07606 (14)	0.0491 (9)	
H3	0.2419	0.5210	0.0878	0.074*	
O4	0.0140 (3)	0.5855 (4)	0.06817 (18)	0.0685 (11)	
C1	0.3850 (3)	0.4087 (6)	0.1657 (2)	0.0411 (11)	
C2	0.3001 (3)	0.4868 (5)	0.20651 (19)	0.0349 (10)	
C3	0.3300 (3)	0.6209 (6)	0.2472 (2)	0.0447 (12)	
H3A	0.4031	0.6573	0.2498	0.054*	
C4	0.2558 (4)	0.7001 (6)	0.2831 (2)	0.0493 (12)	
H4	0.2780	0.7916	0.3095	0.059*	
C5	0.1464 (4)	0.6457 (6)	0.2811 (2)	0.0505 (13)	
H5	0.0950	0.7002	0.3057	0.061*	
C6	0.1159 (3)	0.5112 (6)	0.2422 (2)	0.0438 (11)	
H6	0.0433	0.4714	0.2417	0.053*	
C7	0.1900 (3)	0.4322 (5)	0.20367 (19)	0.0344 (10)	
C8	0.1484 (3)	0.2851 (5)	0.1628 (2)	0.0347 (10)	
C9	0.1496 (3)	0.1076 (6)	0.1828 (2)	0.0442 (12)	
H9	0.1820	0.0787	0.2199	0.053*	
C10	0.1030 (4)	−0.0250 (6)	0.1479 (2)	0.0505 (13)	
H10	0.1074	−0.1447	0.1607	0.061*	
C11	0.0497 (4)	0.0149 (6)	0.0943 (2)	0.0548 (13)	
H11	0.0156	−0.0762	0.0718	0.066*	
C12	0.0474 (3)	0.1894 (6)	0.0745 (2)	0.0466 (12)	
H12	0.0116	0.2177	0.0383	0.056*	
C13	0.0983 (3)	0.3256 (5)	0.1082 (2)	0.0358 (10)	
C14	0.0970 (3)	0.5087 (6)	0.0828 (2)	0.0413 (11)	
C15	0.4040 (3)	0.7289 (6)	0.4267 (2)	0.0461 (12)	
H15	0.4063	0.7534	0.3856	0.055*	
C16	0.3115 (3)	0.6510 (6)	0.4508 (3)	0.0526 (14)	
H16	0.2507	0.6248	0.4263	0.063*	
C17	0.3104 (4)	0.6130 (6)	0.5107 (3)	0.0517 (13)	
H17	0.2485	0.5592	0.5270	0.062*	
C18	0.4003 (3)	0.6533 (5)	0.5482 (2)	0.0417 (11)	
C19	0.4904 (3)	0.7367 (5)	0.5213 (2)	0.0371 (11)	
C20	0.5865 (3)	0.7782 (6)	0.5575 (2)	0.0421 (11)	
C21	0.5874 (4)	0.7370 (6)	0.6186 (2)	0.0503 (13)	
C22	0.6842 (5)	0.7747 (7)	0.6519 (2)	0.0632 (15)	
H22	0.6893	0.7497	0.6930	0.076*	
C23	0.7701 (4)	0.8488 (7)	0.6223 (3)	0.0667 (16)	
H23	0.8354	0.8736	0.6431	0.080*	
C24	0.7609 (4)	0.8868 (7)	0.5623 (2)	0.0568 (14)	
H24	0.8211	0.9386	0.5436	0.068*	
C25	0.4055 (4)	0.6151 (7)	0.6106 (3)	0.0570 (14)	
H25	0.3450	0.5622	0.6287	0.068*	
C26	0.4940 (4)	0.6525 (7)	0.6443 (3)	0.0590 (14)	
H26	0.4951	0.6231	0.6851	0.071*	

### Atomic displacement parameters (Å^2^)

**Table:**

	*U*^11^	*U*^22^	*U*^33^	*U*^12^	*U*^13^	*U*^23^
N1	0.0245 (18)	0.044 (2)	0.046 (3)	−0.0031 (15)	0.0015 (16)	0.0019 (18)
N2	0.033 (2)	0.061 (3)	0.052 (3)	−0.0009 (18)	−0.0029 (18)	0.0035 (19)
O1	0.0283 (16)	0.074 (2)	0.039 (2)	0.0077 (14)	0.0005 (14)	−0.0034 (17)
O2	0.0330 (18)	0.106 (3)	0.063 (3)	0.0231 (18)	−0.0067 (16)	0.001 (2)
O3	0.0361 (17)	0.0474 (18)	0.064 (2)	0.0008 (14)	−0.0022 (15)	0.0129 (16)
O4	0.0416 (19)	0.052 (2)	0.111 (3)	0.0062 (16)	−0.0257 (19)	0.004 (2)
C1	0.026 (2)	0.047 (3)	0.050 (3)	0.0011 (19)	0.005 (2)	0.001 (2)
C2	0.029 (2)	0.037 (2)	0.038 (3)	0.0025 (18)	−0.0028 (18)	0.001 (2)
C3	0.031 (2)	0.053 (3)	0.050 (3)	−0.005 (2)	−0.009 (2)	0.003 (2)
C4	0.056 (3)	0.047 (3)	0.045 (3)	0.007 (2)	−0.009 (2)	−0.008 (2)
C5	0.041 (3)	0.059 (3)	0.051 (3)	0.007 (2)	0.001 (2)	−0.012 (2)
C6	0.032 (2)	0.052 (3)	0.047 (3)	0.004 (2)	0.003 (2)	−0.002 (2)
C7	0.027 (2)	0.038 (2)	0.038 (3)	0.0013 (18)	−0.0018 (18)	0.0033 (19)
C8	0.024 (2)	0.034 (2)	0.046 (3)	0.0014 (17)	0.0036 (19)	−0.004 (2)
C9	0.038 (2)	0.042 (3)	0.053 (3)	0.001 (2)	0.003 (2)	0.001 (2)
C10	0.055 (3)	0.034 (3)	0.063 (4)	0.000 (2)	0.012 (3)	0.001 (2)
C11	0.055 (3)	0.044 (3)	0.065 (4)	−0.010 (2)	0.002 (3)	−0.014 (3)
C12	0.038 (3)	0.043 (3)	0.059 (4)	−0.004 (2)	−0.002 (2)	−0.011 (2)
C13	0.026 (2)	0.037 (2)	0.044 (3)	0.0005 (18)	−0.0017 (19)	−0.001 (2)
C14	0.033 (3)	0.042 (3)	0.049 (3)	0.002 (2)	−0.009 (2)	−0.007 (2)
C15	0.033 (2)	0.051 (3)	0.055 (3)	0.001 (2)	−0.005 (2)	0.000 (2)
C16	0.026 (2)	0.059 (3)	0.073 (4)	−0.007 (2)	0.000 (2)	−0.011 (3)
C17	0.038 (3)	0.046 (3)	0.072 (4)	−0.005 (2)	0.016 (3)	−0.008 (3)
C18	0.038 (3)	0.035 (2)	0.053 (3)	0.0052 (19)	0.013 (2)	−0.001 (2)
C19	0.032 (2)	0.035 (2)	0.044 (3)	0.0055 (18)	0.005 (2)	0.001 (2)
C20	0.039 (3)	0.038 (2)	0.049 (3)	0.011 (2)	0.004 (2)	−0.002 (2)
C21	0.061 (3)	0.045 (3)	0.045 (3)	0.010 (2)	0.000 (2)	0.000 (2)
C22	0.073 (4)	0.072 (4)	0.045 (4)	0.010 (3)	−0.009 (3)	0.002 (3)
C23	0.055 (3)	0.083 (4)	0.062 (4)	0.000 (3)	−0.012 (3)	−0.001 (3)
C24	0.038 (3)	0.073 (4)	0.059 (4)	−0.002 (2)	−0.009 (2)	0.002 (3)
C25	0.055 (3)	0.056 (3)	0.061 (4)	0.007 (3)	0.021 (3)	0.009 (3)
C26	0.068 (4)	0.060 (3)	0.050 (4)	0.015 (3)	0.016 (3)	0.012 (3)

### Geometric parameters (Å, °)

**Table:**

N1—C15	1.324 (5)	C10—H10	0.9300
N1—C19	1.332 (5)	C11—C12	1.363 (6)
N1—H1	0.8600	C11—H11	0.9300
N2—C24	1.324 (6)	C12—C13	1.392 (6)
N2—C20	1.354 (5)	C12—H12	0.9300
O1—C1	1.274 (5)	C13—C14	1.465 (6)
O2—C1	1.221 (5)	C15—C16	1.380 (6)
O3—C14	1.315 (5)	C15—H15	0.9300
O3—H3	0.8200	C16—C17	1.356 (7)
O4—C14	1.197 (5)	C16—H16	0.9300
C1—C2	1.501 (6)	C17—C18	1.395 (6)
C2—C3	1.384 (6)	C17—H17	0.9300
C2—C7	1.399 (5)	C18—C19	1.401 (6)
C3—C4	1.348 (6)	C18—C25	1.412 (7)
C3—H3A	0.9300	C19—C20	1.437 (6)
C4—C5	1.391 (6)	C20—C21	1.390 (7)
C4—H4	0.9300	C21—C22	1.406 (7)
C5—C6	1.361 (6)	C21—C26	1.424 (7)
C5—H5	0.9300	C22—C23	1.359 (7)
C6—C7	1.383 (6)	C22—H22	0.9300
C6—H6	0.9300	C23—C24	1.363 (7)
C7—C8	1.497 (6)	C23—H23	0.9300
C8—C13	1.379 (6)	C24—H24	0.9300
C8—C9	1.384 (6)	C25—C26	1.328 (7)
C9—C10	1.364 (6)	C25—H25	0.9300
C9—H9	0.9300	C26—H26	0.9300
C10—C11	1.376 (7)		
			
C15—N1—C19	122.6 (4)	C8—C13—C14	122.6 (4)
C15—N1—H1	118.7	C12—C13—C14	117.4 (4)
C19—N1—H1	118.7	O4—C14—O3	119.9 (4)
C24—N2—C20	115.8 (4)	O4—C14—C13	123.0 (4)
C14—O3—H3	109.5	O3—C14—C13	117.1 (4)
O2—C1—O1	125.2 (4)	N1—C15—C16	119.9 (5)
O2—C1—C2	118.2 (4)	N1—C15—H15	120.1
O1—C1—C2	116.5 (4)	C16—C15—H15	120.1
C3—C2—C7	118.4 (4)	C17—C16—C15	119.3 (4)
C3—C2—C1	119.5 (4)	C17—C16—H16	120.4
C7—C2—C1	122.1 (4)	C15—C16—H16	120.4
C4—C3—C2	121.7 (4)	C16—C17—C18	121.1 (4)
C4—C3—H3A	119.2	C16—C17—H17	119.4
C2—C3—H3A	119.2	C18—C17—H17	119.4
C3—C4—C5	120.4 (4)	C17—C18—C19	116.9 (5)
C3—C4—H4	119.8	C17—C18—C25	124.2 (5)
C5—C4—H4	119.8	C19—C18—C25	118.9 (4)
C6—C5—C4	118.8 (4)	N1—C19—C18	120.2 (4)
C6—C5—H5	120.6	N1—C19—C20	120.3 (4)
C4—C5—H5	120.6	C18—C19—C20	119.4 (4)
C5—C6—C7	121.7 (4)	N2—C20—C21	124.1 (4)
C5—C6—H6	119.2	N2—C20—C19	116.7 (4)
C7—C6—H6	119.2	C21—C20—C19	119.2 (4)
C6—C7—C2	119.0 (4)	C20—C21—C22	117.4 (5)
C6—C7—C8	117.6 (3)	C20—C21—C26	119.6 (5)
C2—C7—C8	123.4 (4)	C22—C21—C26	123.0 (5)
C13—C8—C9	119.2 (4)	C23—C22—C21	118.1 (5)
C13—C8—C7	120.8 (4)	C23—C22—H22	121.0
C9—C8—C7	119.6 (4)	C21—C22—H22	121.0
C10—C9—C8	119.9 (5)	C22—C23—C24	120.3 (5)
C10—C9—H9	120.1	C22—C23—H23	119.9
C8—C9—H9	120.1	C24—C23—H23	119.9
C9—C10—C11	121.3 (4)	N2—C24—C23	124.4 (5)
C9—C10—H10	119.4	N2—C24—H24	117.8
C11—C10—H10	119.4	C23—C24—H24	117.8
C12—C11—C10	119.2 (4)	C26—C25—C18	122.0 (5)
C12—C11—H11	120.4	C26—C25—H25	119.0
C10—C11—H11	120.4	C18—C25—H25	119.0
C11—C12—C13	120.3 (5)	C25—C26—C21	120.8 (5)
C11—C12—H12	119.8	C25—C26—H26	119.6
C13—C12—H12	119.8	C21—C26—H26	119.6
C8—C13—C12	120.0 (4)		
			
O2—C1—C2—C3	47.2 (6)	C12—C13—C14—O3	−123.8 (4)
O1—C1—C2—C3	−130.0 (4)	C19—N1—C15—C16	−0.1 (6)
O2—C1—C2—C7	−134.7 (5)	N1—C15—C16—C17	−1.3 (7)
O1—C1—C2—C7	48.1 (6)	C15—C16—C17—C18	0.8 (7)
C7—C2—C3—C4	−0.8 (7)	C16—C17—C18—C19	0.9 (6)
C1—C2—C3—C4	177.4 (4)	C16—C17—C18—C25	−179.0 (4)
C2—C3—C4—C5	1.3 (7)	C15—N1—C19—C18	1.9 (6)
C3—C4—C5—C6	0.1 (7)	C15—N1—C19—C20	178.3 (4)
C4—C5—C6—C7	−2.2 (7)	C17—C18—C19—N1	−2.2 (6)
C5—C6—C7—C2	2.7 (7)	C25—C18—C19—N1	177.7 (4)
C5—C6—C7—C8	179.9 (4)	C17—C18—C19—C20	−178.7 (4)
C3—C2—C7—C6	−1.2 (6)	C25—C18—C19—C20	1.2 (6)
C1—C2—C7—C6	−179.4 (4)	C24—N2—C20—C21	1.4 (6)
C3—C2—C7—C8	−178.2 (4)	C24—N2—C20—C19	−177.9 (4)
C1—C2—C7—C8	3.7 (6)	N1—C19—C20—N2	1.9 (6)
C6—C7—C8—C13	84.8 (5)	C18—C19—C20—N2	178.4 (4)
C2—C7—C8—C13	−98.2 (5)	N1—C19—C20—C21	−177.4 (4)
C6—C7—C8—C9	−88.3 (5)	C18—C19—C20—C21	−0.9 (6)
C2—C7—C8—C9	88.7 (5)	N2—C20—C21—C22	−1.2 (7)
C13—C8—C9—C10	1.1 (6)	C19—C20—C21—C22	178.1 (4)
C7—C8—C9—C10	174.3 (4)	N2—C20—C21—C26	−178.5 (4)
C8—C9—C10—C11	−3.2 (7)	C19—C20—C21—C26	0.8 (6)
C9—C10—C11—C12	2.8 (7)	C20—C21—C22—C23	0.0 (7)
C10—C11—C12—C13	−0.1 (7)	C26—C21—C22—C23	177.2 (5)
C9—C8—C13—C12	1.5 (6)	C21—C22—C23—C24	0.9 (8)
C7—C8—C13—C12	−171.6 (4)	C20—N2—C24—C23	−0.4 (7)
C9—C8—C13—C14	−177.7 (4)	C22—C23—C24—N2	−0.7 (8)
C7—C8—C13—C14	9.2 (6)	C17—C18—C25—C26	178.5 (4)
C11—C12—C13—C8	−2.0 (6)	C19—C18—C25—C26	−1.4 (7)
C11—C12—C13—C14	177.2 (4)	C18—C25—C26—C21	1.3 (7)
C8—C13—C14—O4	−124.9 (5)	C20—C21—C26—C25	−1.0 (7)
C12—C13—C14—O4	55.9 (6)	C22—C21—C26—C25	−178.2 (5)
C8—C13—C14—O3	55.4 (6)		

### Hydrogen-bond geometry (Å, °)

**Table:**

*D*—H···*A*	*D*—H	H···*A*	*D*···*A*	*D*—H···*A*
N1—H1···O1^i^	0.86	1.82	2.643 (4)	161
O3—H3···O1	0.82	1.75	2.559 (4)	172

Symmetry codes: (i) −*x*+1, *y*+1/2, −*z*+1/2.
